# Paraphilia and Hypersexuality in Bipolar I Disorder and Histrionic Personality Disorder: A Case Report

**DOI:** 10.7759/cureus.77069

**Published:** 2025-01-07

**Authors:** Thiago M Tavares, Marco A Orsini, Mirela H Magnani, Tuany F Pereira, Bruno Lima Pessôa

**Affiliations:** 1 Family Medicine, Universidade Federal de Santa Catarina, Florianópolis, BRA; 2 Neurology, Universidade Federal do Rio de Janeiro, Rio de Janeiro, BRA; 3 Family Medicine, Universidade do Contestado, Mafra, BRA; 4 Public Health, Universidade Federal de Santa Catarina, Florianópolis, BRA; 5 Neurosurgery, Federal Fluminense University, Niterói, BRA

**Keywords:** bipolar disorder, compulsive sexual behavior, histrionic personality disorder, hysterical personality, paraphilic disorders

## Abstract

Paraphilias are rare and often overlooked psychiatric conditions characterized by intense, atypical, and recurrent sexual urges or behaviors. This case report describes a 33-year-old woman with comorbid bipolar I disorder and histrionic personality disorder, presenting with hypersexuality and risky sexual practices, suggestive of quantitative paraphilia. The patient's behavior included group sexual encounters, unprotected intercourse, and impulsive dissemination of explicit images via social media, culminating in a *Trichomonas vaginalis* infection. Management required an interdisciplinary approach, combining pharmacotherapy (lithium carbonate and quetiapine), cognitive-behavioral therapy (CBT) targeting impulsivity and emotional regulation, and psychoeducational interventions emphasizing safe sexual practices and impulse control.

The report highlights the interconnected nature of severe psychiatric disorders and sexual behavior dysregulation, emphasizing the need for integrated therapeutic strategies to achieve clinical stabilization and minimize risks. This case also underscores gaps in specific guidelines for managing paraphilic behaviors in psychiatric populations, particularly in contemporary sociocultural contexts with evolving sexual dynamics. Future studies are warranted to develop structured, evidence-based approaches to address this complex interplay of psychiatric comorbidities and paraphilias.

## Introduction

Paraphilia refers to sexual arousal linked to stimuli deemed inappropriate, encompassing interests, thoughts, and behaviors outside conventional norms. It does not necessarily require medical intervention or qualify as a mental disorder [1,2]. When associated with psychiatric disorders, such as bipolar affective disorder type I (BAD-I), these conditions can intensify risk behaviors and impair an individual's physical, mental, and social health [3,4]. Hypersexuality, a common feature in manic episodes of bipolar disorder (BD), may be exacerbated by the presence of personality disorders, such as histrionic personality disorder (HPD) [5-7].

Given the relevance of discussing this topic, as well as the scarcity of studies exploring the integrated relationship between bipolar I disorder, HPD, and paraphilias, this study aims to report a clinical case involving these conditions.

This proposal highlights the diagnostic challenges and the interdisciplinary management, considering the gaps in the scientific literature, characterized by greater access to pornography, changes in relationship dynamics, and new expressions of sexual behavior. These factors reinforce the complexity of understanding and addressing the psychopathological conditions under analysis, demanding investigations that transcend the traditional biomedical perspective to encompass emerging cultural and social aspects.

## Case presentation

Clinical history

The patient is a 33-year-old Caucasian woman, unemployed, with a history of unstable relationships, always ending with arguments, each lasting only a few months. She reports that her family does not give her attention and she maintains only minimal contact with her mother. She has a prior diagnosis of BAD-I, based on episodes of excessive spending, euphoric mood, emotional lability, promiscuity, persistent pursuit of social activities or stimuli, verbosity, elevated tone of voice, and hypersexuality, sporadically followed by affective blunting and HPD.

She reported frequent risky sexual behavior over the past two years, with inconsistent use of condoms, and episodes of remorse followed by social withdrawal.

During the reported period (last two months before the consultation), the patient engaged in sexual activities with four men in a single night at a group sex venue, two of them simultaneously. She also reported unprotected sexual intercourse with two men she met at a nightclub, both at the same time. Additionally, she participated in sexual encounters with strangers in public locations, such as bar restrooms, without condom use. Moreover, she sent sexually explicit images to contacts on social media, which often resulted in risky sexual encounters.

A gynecological examination revealed signs of cervicitis, with purulent, foul-smelling, greenish vaginal discharge. Laboratory tests confirmed infection with *Trichomonas vaginalis*. During psychiatric evaluation, based on the Diagnostic and Statistical Manual of Mental Disorders, Fifth Edition, Text Revision (DSM-5-TR) criteria, traits of theatricality and a constant need for attention, characteristic of HPD, were identified.

The patient reported three attempts to access mental health services, with inconsistent adherence to previous treatments, unsuccessful due to her belief of lacking support from friends and family.

Therapeutic management

The therapeutic management involved a multidisciplinary approach. Pharmacological treatment included oral metronidazole 500 mg every 12 hours for seven days for the treatment of trichomoniasis, combined with lithium carbonate 300 mg twice daily for mood stabilization and quetiapine 50 mg at night, adjusted according to clinical response.

Psychotherapy was conducted through cognitive-behavioral therapy (CBT), focusing on impulsivity, emotional regulation, and the reduction of risky behaviors. Psychoeducation sessions emphasized awareness of safe sexual practices, identification of triggers, and the development of strategies for impulse control. Serological tests for syphilis, human immunodeficiency virus (HIV), and viral hepatitis were performed, all with negative results.

Clinical evolution

After one month of treatment, the patient showed significant improvement in gynecological symptoms and better control over impulses during manic episodes, reporting no longer experiencing excessive spending and being able to control her mood and emotions. She reported greater adherence to pharmacological treatment and a better understanding of her psychiatric condition. Low self-esteem and emotional dependence on external validation persisted, requiring continued follow-up.

The patient was advised to avoid alcohol and other psychoactive substances due to potential negative interactions with lithium carbonate and their impact on controlling manic episodes. Quarterly follow-up sessions were scheduled for clinical assessment and therapeutic adjustments.

Continuous use of barrier methods (condoms) and strategies for recognizing emotional triggers were recommended, with reinforcement during psychoeducation sessions.

## Discussion

Relationship between paraphilia and BD

Paraphilia is defined as a pattern of intense and persistent sexual interests involving objects, situations, or individuals outside the conventional context of consensual sexual relationships, often considered socially atypical [1,7]. Paraphilic disorder, in turn, occurs when these interests cause significant distress to the individual or harm to others. For the diagnosis of paraphilic disorder, it is essential that the paraphilia is associated with functional impairment or clinically significant distress [7].

Paraphilias in patients with BD are rarely described in the literature. Manic episodes often exacerbate risky behaviors, including hypersexuality and grandiose goal setting [8-10]. Bipolar patients may exhibit varying degrees of impulsive sexual behavior during manic episodes [4,11]. Approximately 12% of the population presents traits consistent with personality disorders, characterized by inflexible and dysfunctional psychological patterns that contribute to significant functional impairment and intense emotional distress [11,12].

Hypersexuality, often associated with dopaminergic dysfunction, can be aggravated by the presence of personality disorders such as HPD [13,14]. Current scientific literature highlights that bipolar patients frequently face sexual difficulties related to mania-induced hypersexuality and the repercussions of mood cycles on intimate relationships [7,15,16].

However, much of the available research presents methodological limitations, including outdated information and a lack of a uniform definition for the term hypersexuality [4]. Risky sexual behaviors expose patients to sexually transmitted infections, unplanned pregnancies, violence, and life-threatening conditions [17]. In this case, the multidisciplinary approach was crucial for clinical stabilization and minimizing harm associated with impulsive behaviors [13].

Therapeutic strategies and perspectives

Lithium carbonate and quetiapine were effective in controlling manic symptoms, while CBT addressed cognitive distortions related to self-image and external validation. The combination of mood stabilizers and psychotherapy can significantly reduce risky behaviors in bipolar patients [8,14,18,19]. Continuous treatment and psychoeducation are essential to prevent relapses [14].

## Conclusions

This case highlights the complex interaction between bipolar I disorder, HPD, and risky sexual behaviors linked to paraphilias. Impulsivity, emotional instability, and the need for external validation contribute to significant physical, psychological, and social risks. Addressing these behaviors requires an integrated approach, combining medication, psychotherapy, and psychoeducation, tailored to the patient's clinical and cultural context.

The absence of clear guidelines for managing paraphilias in psychiatric patients remains a challenge. This report emphasizes the need for ethical, patient-centered care that ensures safety while respecting autonomy. Further research should focus on developing practical management protocols and evaluating the long-term outcomes of interdisciplinary treatments for such complex cases. Studies exploring tailored therapeutic strategies and preventive interventions in patients with multiple psychiatric comorbidities, such as bipolar I disorder and paraphilias, are crucial to improving treatment outcomes.

## References

[1] (2016). Practical guide to paraphilia and paraphilic disorders.

[2] Sadock BJ, Sadock VA, Ruiz P (2017). Compêndio de psiquiatria: ciência do comportamento e psiquiatria clínica. Artmed, Porto Alegre.

[3] Skodol AE, Grilo CM, Keyes KM, Geier T, Grant BF, Hasin DS (2011). Relationship of personality disorders to the course of major depressive disorder in a nationally representative sample. Am J Psychiatry.

[4] Kopeykina I, Kim HJ, Khatun T, Boland J, Haeri S, Cohen LJ, Galynker II (2016). Hypersexuality and couple relationships in bipolar disorder: a review. J Affect Disord.

[5] Whitbourne SK (2020). Abnormal psychology: clinical perspectives on psychological disorders.

[6] Herpertz SC, Schneider I, Renneberg B, Schneider A (2022). Patients with personality disorders in everyday clinical practice-implications of the ICD-11. Dtsch Arztebl Int.

[7] (2022). Diagnostic and Statistical Manual of Mental Disorders, Fifth Edition, Text Revision (DSM-5-TR). https://psychiatryonline.org/doi/book/10.1176/appi.books.9780890425787.

[8] Goodwin FK, Jamison KR (2007). Manic-depressive illness: bipolar disorders and recurrent depression. Am J Psychiatry.

[9] Johnson SL, Carver CS (2006). Extreme goal setting and vulnerability to mania among undiagnosed young adults. Cognit Ther Res.

[10] Kafka MP (2010). Hypersexual disorder: a proposed diagnosis for DSM-V. Arch Sex Behav.

[11] Semple D, Smyth R (2019). Oxford handbook of psychiatry. Oxford Handbook of Psychiatry. 4th Edition.

[12] Widiger TA (2012). The Oxford handbook of personality disorders.

[13] Barlow DH, Durand VM, Hofmann SG (2018). Abnormal psychology: an integrative approach. https://archive.org/details/barlow-david-h-durand-v-mark-hofman-stefan-g-abnormal-psychology/page/n1/mode/2up.

[14] Eysenck MW (2022). Simply psychology. https://search.worldcat.org/title/1266205777.

[15] Stahl SM (2020). Prescriber's guide: Stahl's essential psychopharmacology.

[16] Belmaker RH (2007). Treatment of bipolar depression. N Engl J Med.

[17] Rossegger A, Bartels RM, Endrass J, Borchard B, Singh JP (2021). High risk sexual fantasies and sexual offending: an overview of fundamentals and interventions. Sex Offending.

[18] Cordioli AV, Gallois CB, Passos IC (2023). Psicofármacos: consulta rápida.

[19] Miguel EC, Lafer B, Elkis H, Forlenza OV (2021). Clínica psiquiátrica: a terapêutica psiquiátrica.

